# Extraction of tamoxifen and its metabolites from formalin-fixed, paraffin-embedded tissues: an innovative quantitation method using liquid chromatography and tandem mass spectrometry

**DOI:** 10.1007/s00280-013-2346-z

**Published:** 2014-01-12

**Authors:** Ella S. M. Ng, S. Bill Kangarloo, Mie Konno, Alexander Paterson, Anthony M. Magliocco

**Affiliations:** 1 Pharmacokinetic Unit, Tom Baker Cancer Centre, Alberta Health Services, Calgary, AB Canada; 2 Department of Pathology and Oncology, University of Calgary, Calgary, AB Canada; 3 Department of Anatomical Pathology, H. Lee Moffitt Cancer Centre and Research Institute, 12902 Magnolia Drive, Tampa, FL 33612 USA

**Keywords:** Tamoxifen therapy, Breast cancer recurrence, Retrospective analysis, Formalin-fixed paraffin-embedded tissue, Tandem mass spectrometry

## Abstract

**Purpose:**

Tamoxifen is a key therapeutic option for breast cancer treatment. Understanding its complex metabolism and pharmacokinetics is important for dose optimization. We examined the possibility of utilizing archival formalin-fixed paraffin-embedded (FFPE) tissue as an alternative sample source for quantification since well-annotated retrospective samples were always limited.

**Methods:**

Six 15 μm sections of FFPE tissues were deparaffinized with xylene and purified using solid-phase extraction. Tamoxifen and its metabolites were separated and detected by liquid chromatography–tandem mass spectrometry using multiple-reaction monitoring.

**Results:**

This method was linear between 0.4 and 200 ng/g for 4-hydroxy-tamoxifen and endoxifen, and 4–2,000 ng/g for tamoxifen and *N*-desmethyl-tamoxifen. Inter- and intra-assay precisions were <9 %, and mean accuracies ranged from 81 to 106 %. Extraction recoveries were between 83 and 88 %. The validated method was applied to FFPE tissues from two groups of patients, who received 20 mg/day of tamoxifen for >6 months, and were classified into breast tumor recurrence and non-recurrence. Our preliminary data show that levels of tamoxifen metabolites were significantly lower in patients with recurrent cancer, suggesting that inter-individual variability in tamoxifen metabolism might partly account for the development of cancer recurrence. Nevertheless, other causes such as non-compliance or stopping therapy of tamoxifen could possibly lead to the concentration differences.

**Conclusions:**

The ability to successfully study tamoxifen metabolism in such tissue samples will rapidly increase our knowledge of how tamoxifen’s action, metabolism and tissue distribution contribute to breast cancer control. However, larger population studies are required to understand the underlying mechanism of tamoxifen metabolism for optimization of its treatment.

## Introduction

Tamoxifen (TAM) has an important role in breast cancer treatment and prevention. This drug inhibits breast cancer by competing with endogenous estrogens for binding to estrogen receptors (ERs) in tumor tissue. Despite the proven anti-cancer effects of TAM, numerous studies have shown that the properties of this drug may cause detrimental effects such as increased risk of endometrial cancer and thromboembolic diseases [1–4]. Moreover, many patients develop resistance to TAM therapy after several years of treatment and eventually experience cancer recurrence [5–7]. The therapeutic and adverse effects of TAM have stimulated interest in understanding the biological activity of this drug. Indeed, there is a growing body of evidence indicating that the anti-proliferative effects of TAM depend primarily on the formation of its active metabolites.

In the primary metabolic pathway, TAM is metabolized in the liver by cytochrome P450 enzymes CYP3A4/5 and CYP2D6 to *N*-desmethyl-tamoxifen (DMT) and 4-hydroxy-tamoxifen (4-OH), respectively. Through CYP3A4/5, 4-OH is converted to *N*-desmethyl-4-hydroxy-tamoxifen (endoxifen), whereas DMT is further metabolized to endoxifen via CYP2D6 [8–10]. Studies have shown that 4-OH and endoxifen have much higher affinity to ERs and are more effective in suppressing estrogen-dependent breast cancer than the parent drug [10–13]. Many researchers have suggested that the effectiveness of TAM therapy depends highly on the functionality of CYP2D6 because polymorphisms or drug-induced alterations of this gene may lead to reduced enzyme activity and consequently result in lower concentrations of 4-OH and endoxifen, thus affecting the drug efficacy [14–16]. Apparently, the inter-individual differences in metabolism may be associated with the variability in TAM response [9, 17]. Although pharmacogenetic testing of CYP2D6 polymorphisms has been recommended to identify patients with poor responsiveness to TAM [18], recent studies suggest that CYP2D6 genotyping could not fully predict the metabolite concentrations for an evaluation of the drug efficacy [15, 21]. Indeed, direct measurement of the active metabolites and therapeutic monitoring of their levels were recommended to be considered during the TAM therapy [19–21]. Clearly, these studies suggest that quantitative measurement of TAM metabolites is crucial in understanding the TAM metabolism for a comprehensive evaluation of its therapy.

In the past, plasma or fresh/frozen tissue samples have been the main source for drug and other small molecule analysis. Nevertheless, obtaining these samples for clinical testing is often problematic due to lack of well-annotated retrospective samples taken from patients undergoing TAM therapy. In this regard, our laboratory had been seeking alternative sample sources and inquired whether the quantities of formalin-fixed, paraffin-embedded (FFPE) tissues that are regularly prepared from biopsies and at autopsy and stored in the archives of the hospital could be used for quantitation of TAM and its metabolites in search of clinically and biologically important information.

In this pilot study, we examined the feasibility of extracting TAM and its metabolites from the archival FFPE tissue blocks. We have developed and validated a liquid chromatography–tandem mass spectrometry (LC–MS/MS) method utilizing solid-phase extraction (SPE) for sample preparation from FFPE tissue. The extracted analytes are detected by a highly sensitive triple-quadrupole mass spectrometer in positive electrospray ionization mode using multiple-reaction monitoring (MRM). This methodology not only demonstrates the possibility of simultaneous quantitation of TAM and its metabolites, but also facilitates the development of small molecule extraction from FFPE tissue. More importantly, this assay provides an opportunity for researchers to excavate valuable information from FFPE tissue, especially when these archival samples are the only source of biomaterial available.

## Materials and methods

### Chemicals and reagents

Tamoxifen, tamoxifen-d5, 4-hydroxy-tamoxifen (pure *Z*-trans-isomer, >98 % *Z*), 4-hydroxy-tamoxifen-d5, *N*-desmethyl-tamoxifen hydrochloride, *N*-desmethyl-tamoxifen-d5, *N*-desmethyl-4-hydroxy-tamoxifen (1:1 *E*/*Z* mixture) and *N*-desmethyl-4-hydroxy-tamoxifen-d5 (1:1 *E*/*Z* mixture) were obtained from Toronto Research Chemicals (North York, ON, Canada). Optima-grade acetonitrile, methanol, water and certified ACS xylene were purchased from Fisher Scientific (Fair Lawn, NJ, USA). Formic acid and ammonium acetate were from Sigma-Aldrich (Oakville, ON, Canada). Bond Elut^®^ C2 (100 mg, 3 mL) solid-phase extraction (SPE) cartridges were obtained from Agilent Technologies (Santa Clara, CA, USA).

### Preparation of standard curves, internal standards and quality controls

Stock solutions of 4-OH, endoxifen, TAM and DMT were prepared individually at 1 mg/mL in methanol. A mixture of working standards was prepared by diluting the stock solutions in methanol at 0.1–100 ng/mL for 4-OH and endoxifen, and 1–1,000 ng/mL for TAM and DMT. Standard curves were prepared by spiking known concentrations of each mixture to non-TAM FFPE tissues (paraffin tissue blocks from patients prior to TAM treatment). Concentrations were corrected for tissue mass (~25 mg) and expressed in nanogram of TAM or metabolites per gram of breast tumor tissue extracted from the tissue blocks. The concentration ranges for the standard curves were 0.4–200 ng/g for 4-OH and endoxifen, and 4–2,000 ng/g for TAM and DMT.

Deuterated internal standards (I.S.) were used for quantitation since they closely resembled the analytes in extraction and chromatographic properties, and also corrected for the loss of analytes during sample preparation. A mixture of I.S. working solution consisting of tamoxifen-d5, 4-hydroxy-tamoxifen-d5, *N*-desmethyl-tamoxifen-d5 and *N*-desmethyl-4-hydroxy-tamoxifen-d5 was prepared by diluting each individual stock solution (1 mg/mL) in methanol to a concentration of 50 ng/mL. A final concentration of 10 ng/mL (equivalent to 400 ng/g per analysis) was added to each FFPE tissue sample.

In-house quality controls (QC) were prepared at 100 ng/g for 4-OH, endoxifen and 1,000 ng/g for TAM, DMT in non-TAM FFPE tissues. The QC was processed with each batch of paraffin tissue samples to assess the accuracy of the analytical method.

### Patients and FFPE tissue samples

This study was approved by the Conjoint Health Research Ethics Board. FFPE tissue blocks were acquired from the archives of the pathology department at Foothills Medical Centre (Calgary, AB, Canada). Patients who had estrogen-receptor positive breast cancers diagnosed from 1990 to 2010 and treated with standard doses of TAM (20 mg/day of TAM for at least 6 months until time of biopsy with a treatment plan of 5 years on TAM) were selected for investigation. Patients were divided into two groups categorized as breast tumor recurrence (*n* = 16) and non-recurrence (*n* = 8). Non-recurrent breast cancer patients were classified as those who developed suspicious breast lesions during their follow-up visit after several months of TAM treatment but had a benign pathology diagnosis upon breast tissue biopsy (range 6–26 months).

### Sample preparation

Six 15 μm paraffin sections were removed from each FFPE tissue block and placed in a 1.5 mL eppendorf tube. The tissue sections were then deparaffinized by incubating with 1 mL of xylene for 10 min at room temperature. About 20 μL of I.S. was added followed by centrifugation at 20,000*g* for 5 min. Since xylene washes most of the TAM and metabolites from the paraffin sections, the supernatant was collected and transferred to a clean eppendorf tube. This procedure was repeated, and the two supernatants were combined followed by an SPE clean-up. Sample purification was performed via an SPE column vacuum manifold (Supelco, PA, USA). Samples were loaded onto the SPE columns previously conditioned with 3 mL of methanol and acetonitrile followed by a wash with 2 mL of 10 % methanol. The SPE cartridges were dried thereafter for 2 min, and the analytes were eluted with 2 mL of 5 % 50 mM ammonium acetate in methanol. Eluates were evaporated to dryness under a stream of nitrogen at 55 °C using a heating module obtained from Thermo Scientific/Pierce (Asheville, NC, USA). The dry extracts were then resuspended in 100 μL of acetonitrile/0.2 % formic acid (50:50).

### LC–MS/MS analysis

Liquid chromatography was performed on a 1200 SL series LC system consisting of a binary pump, well-plate autosampler and thermostated column compartment (Agilent Technologies). Separation of TAM and metabolites was carried out using a Zorbax SB-C18 column (150 × 2.1 mm i.d., 3.5 μm particle size, Agilent Technologies) with a column temperature of 40 °C. Mobile phase consisted of 0.2 % formic acid and acetonitrile (60:40) at a flow rate of 0.2 mL/min with a gradient of 40–90 % of acetonitrile over 5 min. About 30 μL of the sample extract was injected onto the LC system. The total run time was 12 min including column equilibration.

MS detection of the analytes was accomplished by a 6410 triple-quadrupole mass spectrometer equipped with an electrospray ion source operating in the positive ion mode (Agilent Technologies). High purity nitrogen was used as the drying gas at a flow rate of 11 L/min with a gas temperature of 350 °C, and the nebulizer pressure was set at 35 psi. The capillary voltage was set at 4,000 V for each compound; however, the fragmentor voltages and the collision energies were operated at different settings for optimization of each analyte (Table 1). Quantitation was performed in multiple-reaction monitoring (MRM) mode, and the transitions of the precursors to product ions are summarized in Table 1. Data acquisition and analysis were performed using Mass Hunter Software v.B.02.01 (Agilent Technologies).

**Table 1:** MRM transitions and MS operating parameters for tamoxifen, the three metabolites and their corresponding I.S

Compounds	MRM transitions	Fragmentor (V)	Collision energy (V)
TAM	372 → 72	140	25
TAM-d_5_	377 → 72	140	25
DMT	358 → 58	120	20
DMT-d_5_	363 → 58	120	20
4-OH	388 → 72	140	25
4-OH-d_5_	393 → 72	140	28
Endoxifen	374 → 58	120	22
Endoxifen-d_5_	379 → 58	120	22

### Validation procedures

#### Linearity

Standard curves for TAM and the three metabolites were obtained by linear regression analysis using internal standardization. Seven-point standard curves in the range of 0.4–200 and 4–2,000 ng/g were constructed for 4-OH/endoxifen and TAM/DMT, respectively. The linearity for each compound was determined by plotting the peak area ratio of the analyte to I.S. versus the concentration. The standard curves were generated from three independent runs. The correlation coefficients (*R* ^2^) of the slope of the curves were examined and considered to be acceptable if greater than 0.975.

#### Specificity and sensitivity

Non-TAM FFPE tissue samples spiked at the lowest limit of quantitation (LOQ) and QC levels were extracted and compared to the blank samples (no spiked analytes, with I.S. only). Limit of detection (LOD) for this assay was determined by a serial dilution of the highest calibration point until the lowest detectable concentration was reached.

#### Precision and accuracy

Intra-assay accuracy and precision were determined by analyzing 5 replicates of 3 different levels of concentrations at 4, 40 and 100 ng/g for 4-OH/endoxifen, and 40, 400 and 1,000 ng/g for TAM/DMT in a single LC–MS/MS run, while inter-assay data were collected by running the aforementioned spiked samples on three different days together with linearity. The concentrations of each sample were calculated using the standard curves, and the percent coefficients of variation (%CV) were examined. Accuracy was calculated as the differences between the measured and the theoretical concentration.

#### Extraction efficiency

Extraction recoveries of the four compounds were determined as follows: FFPE tissues were spiked at 40 ng/g for 4-OH/endoxifen and 400 ng/g for TAM/DMT and submitted to extraction as described above. The dried samples were spiked with the I.S. in mobile phase. The mean peak area ratios of the three processed samples were compared to the un-extracted samples, where the analytes and I.S. were spiked after SPE. The results were expressed as percent recoveries (% recovery) = (extracted/unextracted) × 100.

#### Carry-over

Carry-over was determined by running a blank solvent and three non-TAM FFPE tissue samples (with primary breast tumor before TAM treatment started) after the highest calibrator (200 ng/g for 4-OH/endoxifen and 2,000 ng/g for TAM/DMT). A QC was also injected after the samples to ensure proper assay performance.

### Statistical analysis

All experimental data were processed using Microsoft^®^ Excel 2007 software and expressed as mean ± SEM. The coefficient of variation (CV) was expressed as the ratio of the standard deviation to the mean.

Statistical analysis was performed using two-tail unpaired Student’s *t* test at 95 % confidence interval (GraphPad Prism v.2.0) for comparison between the two groups of breast cancer patients. All results were considered significant at *p* values of less than 0.05.

## Results and discussion

### Linearity

The assay was linear over the range of 0.4–200 ng/g for 4-OH/endoxifen and 4–2,000 ng/g for TAM/DMT. Mean correlation coefficients (*R* ^2^) were between 0.9995 and 0.9999 (*n* = 3). The slopes of the standard curves for each compound were consistent with coefficient of variation (CV) between 2.1 and 8.7 % (data not shown). Quantitation of the analytes in FFPE tissues obtained from TAM-treated patients was achieved using peak area ratios of each individual compound to I.S. and was interpolated from the standard curves.

### Specificity and sensitivity

**Fig. 1:**
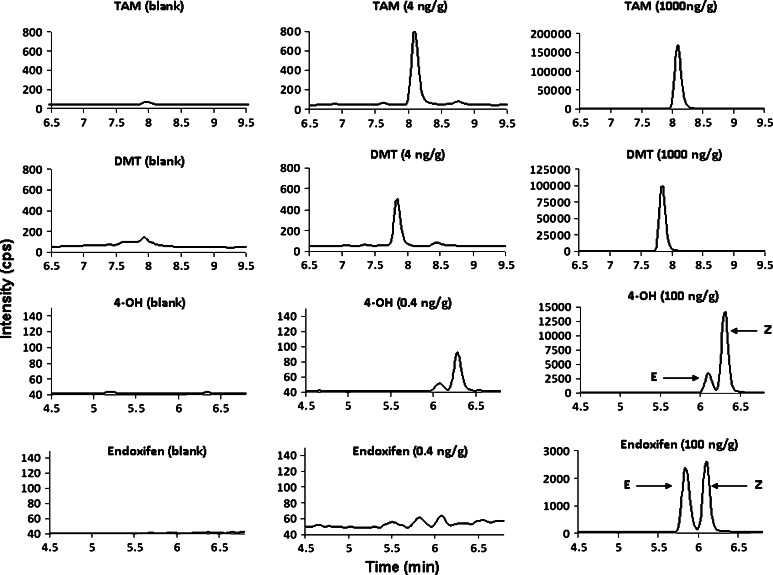
Representative MRM chromatograms of each analyte spiked at LOQ (0.4 ng/g for 4-OH and endoxifen and 4 ng/g for TAM and DMT, 2nd column panel) and QC levels (100 ng/g for 4-OH and endoxifen and 1,000 ng/g for TAM and DMT, 3rd column panel) in non-TAM FFPE tissues, and their corresponding blanks (1st column panel)

The MRM technique used in our LC–MS/MS assay provides a high degree of sensitivity and specificity. Interference from substances other than those being analyzed is not likely to occur due to the choice of specific parent–daughter fragmentation ion and retention time monitoring. Figure 1 demonstrates that TAM and its metabolites spiked in non-TAM FFPE tissues at the LOQ and QC levels were highly distinguishable from the non-spiked. The LOQ of our assay was set at 4 ng/g for TAM and DMT, but 0.4 ng/g was used as the lowest quantitation point for 4-OH and endoxifen since these two metabolites have lower concentration in vivo [20]. LOD of the assay was obtained based on the concentrations that produced a signal-to-noise (*S*/*N*) ratio of ≥3 to deduce the presence of the analytes in FFPE tissue. Our results show that 2 ng/g was the LOD for TAM and DMT, whereas 0.2 ng/g was observed for 4-OH and endoxifen (data not shown).

It should be noted that two chromatographic peaks were observed for endoxifen in the spiked FFPE tissue because the reference compound of endoxifen was a racemic mixture of *E*-endoxifen and *Z*-endoxifen. Additionally, a small peak of *E*-4-OH was identified probably due to the impurity of cis-isomer in the standard (Fig. 1). Although the peaks were well resolved with different retention time in our assay, only *Z*-endoxifen and *Z*-4-OH were quantitated since the drug given to patients was a trans-isomer of *Z*-TAM. Also, previous studies have shown that *Z*-isomers of endoxifen and 4-OH had stronger ER activity than *E*-isomers (*E*-isomers are weak agonists with <1 % of the affinity to the ER as compared to the *Z*-isomers) [22–25], and thus, the focus of this study was primarily on the *Z*-isomers as they are considered as the major active metabolites in TAM treatment.

### Precision and accuracy

Table 2 summarizes the results for precision and accuracies for each compound. Our results show that this assay has good intra- and inter-day precision since the %CV are less than 10 %. Additionally, the accuracies of each analyte were within ±20 % of the true concentrations.

**Table 2:** Validation data for intra- (*n* = 5) and inter-assay (*n* = 3) precision and accuracy

Analyte (concentration in ng/g)	Intra-assay precision (%CV)	Intra-assay accuracy (%)	Inter-assay precision (%CV)	Inter-assay accuracy (%)
*TAM*				
40	2.56	80.5	5.94	100
400	2.40	95.4	4.94	106
1,000	1.58	104	4.54	102
*DMT*				
40	3.27	83.3	8.96	102
400	3.34	92.1	1.96	106
1,000	1.44	99.9	2.09	99.6
*4-OH*				
4	3.36	87.3	6.56	101
40	2.78	98.0	1.80	106
100	3.72	103	0.661	100
*Endoxifen*				
4	3.86	86.9	5.66	82.5
40	2.47	93.2	0.710	105
100	2.62	97.5	3.19	95.3

### Extraction efficiency

The determination of extraction efficiencies for each analyte was performed in triplicates. Recoveries were good and consistent, ranging between 83 and 88 %.

### Carry-over

TAM and its metabolite peaks were not observed in either the blank solvent or the non-TAM FFPE tissue samples after running the high concentration standard, and the calculated concentration of the QC was within ±20 % of the expected value.

### Recovery of TAM and metabolites from FFPE tissue matrix

Formalin fixation with paraffin embedding is the standard procedure for tissue preservation and stabilization in pathology laboratories prior to histological evaluation by pathologists. Previous studies have suggested that the fixation, paraffin processing conditions are harsh, and the prolonged FFPE tissue storage times have made retrospective biological studies difficult since the efficiency of protein recovery was influenced by the fixation protocols, fixation time and sample age [26, 27]. However, numerous groups have demonstrated that the quantity and quality of proteins identified by LC tandem MS from FFPE tissue samples are not significantly impacted by fixation and tissue processing when compared with matched frozen tissues [28–30]. In addition, the study from Scicchitano et al. [31] showed that the number of proteins extracted from FFPE tissue was, indeed, more than the frozen samples. Furthermore, Crockett et al. [32] demonstrated similar results of protein yields, and Palmer-Toy et al. [33] reported a 30 % greater yield of identified proteins from FFPE tissue compared with frozen samples. Clearly, the bulk of evidence would support the view that formalin fixation did not necessarily affect protein recovery and molecules that are bound to the proteins.

In the current study, TAM and its metabolites are the target analytes that bound to proteins in FFPE tissue. Apparently, ethanol that was used as a dehydrating agent in preparation for paraffin wax infiltration did not fully deplete TAM and its metabolites from the tumor tissues because these molecules are hydrophobic; they tend to bind to proteins and remain in the resin until xylene was added to the FFPE tissues. Xylene, which acts as a tissue clearing agent following dehydration, is non-polar and paraffin miscible. This solvent dissolves the paraffin and extracts lipids, proteins as well as the non-polar analytes from the tissues. Once the tissues were spun down, all the hydrophobic components remain in the organic solvent as supernatant.

Although it is noteworthy to compare both archival samples and frozen tissues from the same individual, for our archival samples this option was not available. Nevertheless, our results show that the mean concentrations of TAM, 4-OH, endoxifen and DMT extracted from FFPE tumor recurrent tissues were 122 ± 25.2, 2.13 ± 0.604, 19.2 ± 5.08 and 324 ± 103 ng/g, respectively (Table 3). Our findings are comparable with those of Furlanut et al. [34], who showed that frozen tissues (~2 mg) in breast cancer patients receiving a single dose of 20 mg of TAM (samples were taken 5 h postdrug administration) contained an average concentration of 118 ng/g TAM, 4.90 ng/g 4-OH and 23.3 ng/g DMT (ranging from 0 to 643 ng/g for DMT) (no data for endoxifen). Our data demonstrate that TAM and its metabolites were still recovered from FFPE tissues despite multiple uses of organic solvents for washing and removal of formalin during sample processing.

**Table 3:** Mean concentrations of TAM and its metabolites in tumor recurrent and non-recurrent patients. Mean values of body mass index (BMI) and duration of TAM treatment for each group of patients are demonstrated in the table below

	TAM (ng/g)	DMT (ng/g)	4-OH (ng/g)	Endoxifen (ng/g)	BMI	Months on TAM
Tumor recurrent patients (*n* = 16)	122 ± 25.2	324 ± 103	2.13 ± 0.604	19.2 ± 5.08	26.4	26
Tumor non-recurrent patients (*n* = 8)	147 ± 51.7	1,256 ± 282.8	9.75 ± 4.62	94.6 ± 41.0	28.6	22

To verify whether TAM and its metabolites were fully extracted from the de-waxed tumor samples, FFPE tissue spiked with the analytes was homogenized with methanol after deparaffinization followed by solid-phase extraction. Our results show that only 3–4 % of TAM and its metabolites were recovered from the homogenized tissues comparing to a spiked solvent extracted under the same conditions (data not shown), suggesting that most analytes from the tissue went into the xylene fraction during the deparaffinization process. Similarly, the tissue homogenates and the xylene fractions from the breast tumor patients were tested to ensure optimal extraction efficiency.

### Patient sample analysis

**Fig. 2:**
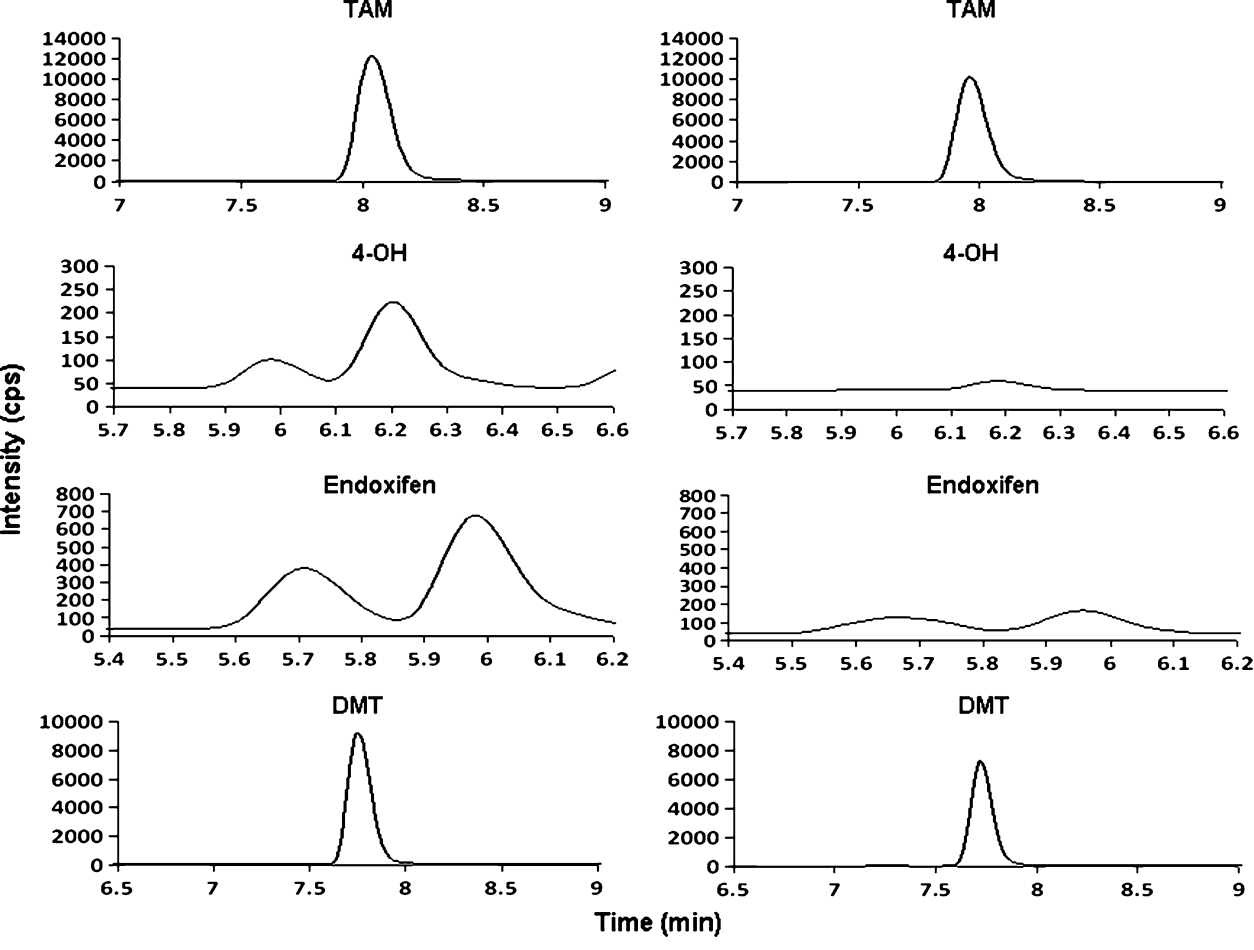
Representative MRM chromatograms of TAM, 4-OH, endoxifen and DMT from a tumor non-recurrent sample (*left panel*) and a recurrent specimen (*right panel*)

**Fig. 3:**
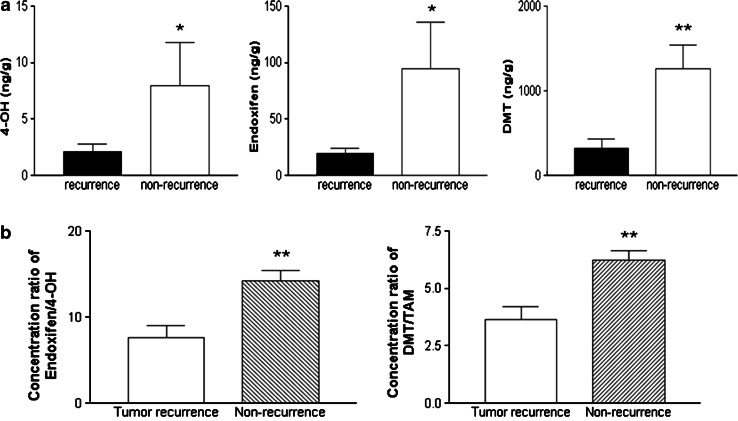
**a** Concentrations of 4-OH (*top left*), endoxifen (*top middle*) and DMT (*top right*) in tumor recurrent and non-recurrent patients. Data are presented as mean ± SEM. The concentrations of each metabolite were significantly higher in patients without breast tumor recurrence. *p < 0.05 relative to tumor recurrence for 4-OH and endoxifen. **p < 0.01 for DMT. **b** The concentration ratios of endoxifen to 4-OH (*bottom left*) and DMT to TAM (*bottom right*) for two groups of breast cancer patients. The ratios for patients without tumor recurrence were significantly higher comparing to the recurrent group. Data are presented as mean ± SEM. **p < 0.01 relative to tumor recurrence

FFPE tissues from breast cancer patients obtained in the archives of the pathology department were investigated to demonstrate the applicability of the assay. Figure 2 contains representative MRM chromatograms of a single tissue specimen from a patient without tumor recurrence comparing to one taken from a patient developing recurrence. It should be noted that only the absolute abundances of TAM and its metabolites are shown in Fig. 2 to illustrate the level of each compound found in the two groups of patients. The true recovery of the analytes in the sample was normalized by the I.S. (data not shown) as area ratios and the concentrations were calculated with respect to the standard curves. Indeed, Table 3 summarizes the mean concentrations of TAM (147 ± 51.7 ng/g), 4-OH (9.75 ± 4.62 ng/g), endoxifen (94.6 ± 41.0 ng/g) and DMT (1,256 ± 282.8 ng/g) in patients without tumor recurrence, whereas in tumor recurrent patients, the average concentrations were 122 ± 25.2, 2.13 ± 0.604, 19.2 ± 5.08 and 324 ± 103 ng/g for TAM, 4-OH, endoxifen and DMT, respectively. Interestingly, the levels of the TAM metabolites were significantly higher in tumor non-recurrent patients (*p* < 0.05 for 4-OH and endoxifen, and *p* < 0.01 for DMT) than those with tumor recurrence (Fig. 3a). One could argue that the concentration difference between the two groups of patients could be associated with the dosage problem of TAM because they were given 20 mg/day of TAM regardless of their weights. Presumably, the levels of metabolites found in each patient should be comparable if TAM was dosed accurately. It is speculated that heavier patients might not have received enough dose of TAM, thereby producing lower levels of metabolites. Also, the duration of TAM treatment before biopsy could affect the levels of metabolites. A longer period of TAM treatment could lead to higher concentrations of metabolites found in the tumor non-recurrent patients. However, our study shows that the duration of TAM treatment for the two groups of patients was on average 26 and 22 months for tumor recurrent and non-recurrent patients, respectively. Moreover, the mean values of body mass index (BMI) for recurrent patients were 26.4 versus 28.6 for non-recurrent subjects, suggesting that a dosing problem of TAM was very unlikely (Table 3) because the length of treatment (*p* = 0.2572) and the patients’ BMI (*p* = 0.4260) were not significantly different. Another interesting phenomenon observed in our study is that 4-OH and endoxifen were not found in some of the tumor recurrent patients (data not shown), suggesting that TAM was possibly not metabolized in these patients or below the detection limit of the assay.

To further investigate whether the P450 isoforms from each group of patients could possibly affect the levels of TAM metabolites, the concentration ratios of each analyte from the two groups were plotted. Figure 3b demonstrates that the concentration ratios of endoxifen to 4-OH and DMT to TAM were significantly different between the two groups (*p* < 0.01), suggesting that individual differences in TAM metabolism may contribute to the development of cancer recurrence and could be attributed to inter-individual variation in cytochrome P450 activity. However, it should be stressed that patients who had tumor recurred might not comply to take tamoxifen. In our experience, by 5 years only 50–60 % of patients still take the drug even though it is prescribed. Also, patients who recurred and had biopsies might have stopped taking tamoxifen or tamoxifen might have been stopped and substituted by another hormone even before biopsy confirmation. Clearly, unknown possibility such as non-compliance or therapy termination could be a cause of the concentration differences.

## Conclusions and future directions

In this study, we have demonstrated the possibility of extracting TAM and its metabolites from FFPE tissue. While there is literature on these analytes in plasma and breast tumor frozen tissue using LC–MS/MS methodologies, to the best of our knowledge, this is the first assay that allows quantitation of small molecules from archival paraffin tissues using targeted LC–MS/MS. Our preliminary data show that the levels of TAM metabolites were significantly higher in tissues taken from patients who did not develop recurrence compared to those who did, suggesting that inter-individual differences in TAM metabolism could account for the development of cancer recurrence. Nevertheless, larger population studies are required to understand the underlying mechanism of TAM metabolism for optimization of its treatment. In our future studies, correlation of levels in FFPE tissue to those in plasma will be undertaken in the hope of increasing our knowledge of how TAM’s action, metabolism and tissue distribution could contribute to breast cancer control. We believe in circumstances where impaired drug metabolism is suspected in a patient; the ability to directly identify non-metabolized molecules in patient biosamples such as routine tissue biopsies and blood samples may be a better alternative or complementary to pharmacogenomic approaches since genetic variations or multiple drugs taken by the patients may inhibit the enzyme activity of CYP2D6 or CYP3A4/5 that interferes with the TAM metabolic pathways, which will not be detected by genomic approaches alone. The methodologies presented here will be a valuable tool in searching for clinically important information, such as correlation of side effects and toxicities with TAM metabolite levels, especially when archival samples represent the only source of biomaterial available. The success of this proof of concept study may encourage investigations into the possibility of direct measurement of other similar drugs, metabolites, hormones and toxins in archival FFPE tissues.
